# 

**Published:** 1855-01

**Authors:** 


					﻿CONTENTS.
PAGE.
ORIGINAL COMMUNICATIONS:—
A Lecture on the Effects of Alcoholic Drinks on the Human System, and
the Duties of Medical Men in Relation thereto. By N. S. Davis, M.D.,.. .97
Amputation of the Leg..................................................124
Disease of the Heart. By R. S. L.......................................125
Notes of Experiments on Ulcers and Wounds. By Daniel Brainard, M. D., 145
Nitrate of Silver as a Local Remedy in Phlegmasia Dolena, By J. R.
Mills, M. D,........................................................150
Chloroform; its Uses, etc. By P. A. Allaire, M. D......................151
Cases from the Note Book. By Owen Long, M. D...........................591
Report of a Case ofPeurperal Convulsions, with remarks on the use of Chlo-
roform. By William Clarke, M. D.,................................£.....193
Monthly Report on the Sanitary Condition of Chicago and the Progress of
Epidemics. By N. S. Davis, M-D.,.......................................198
Uterine Polypus. By George Higgins, M.D.,..............................205
Hints to Young Practitioners on the application of certain Bandages, &c.
By B. E. Dodson, M. D.,............................................  207
Chloroform, its effects, etc. By P. A. Allaire, M. D.,.................241
A case of Strangulation of the Posterior Lip of the Os Tincae, within the
superior aperture of the Pessary. By C. Goodbrake, M. D., Clinton, Ill., 249
Cured by extracting a Tooth. By C. A. Hathwell, M.D.,..................251
The Medical Properties and Use of Iodine. By F. R. Payne, M. D., Mar-
shall, Ill., ........................................................  297
A case of Injury of the knee-joint, with successful treatment,.........302
Adherent Placenta, Profuse Homorrhage, Compression of the Aorta, etc.
By Ira E. Oatman, Sacramento,........................................804
Snake-bite treated by Brandy. By J. R. Thomas, Monticello, Ind.,.......305
Report of a case of Scrofulous Caries of the Head, etc.,...............307
Report of the Sanitary Committe of Cook county Med. Society,...........309
The Pathology of Fevers. (Continued). ByN. S. Davis,...................313
Proceedings of the JEsculapian Society,................................321
Puerperal Convulsions. By T. D. Washburn, M. D.,.......................345
Carcinoma of the Uterus, complicated with Universal Dropsy. By H.
Ritchie, M. D., of Chicago,..........................................351
Death caused by the Injudicious use of Antimony. By C. W. Le Boutillier, 353
A case of Diabetes,....................................................356
Homoeopathy, its Principles and Practice.	By	C. A. Hathwell,..........357
A case of Eclampsia. By D. W. Stormout,	M.	D.,.......................393
A Case. By G. W. Parkins, M. D.,.......................................398
Pathology of Fevers. By N. S. Davis, M. D.,............................401
A case of Twins, with Malpresentation,.................................441
Proceedings of the Cook county Medical Society,........................443
A case of Lunacy,......................................................445
A case of Inverted Uterus,.............................................447
The Lake Superior Region Favorable to Health and Longevity,............450
Case of Eczema,........................................................489
Report on Sanitary characteristics of Chicago,.........................495
SELECTIONS:—
A New Broth for the Sick,..............................................164
Uses of the Miscroscope in the Diagnosis of Cancer,....................165
A Case of Vertebral Dislocation with Favorable Termination,............167
Indications for Treatment in Fever afforded by the state of the Heart,.... .169
PACK
Case of Gastrotoiny for the Removal of the Leaden Bar,...................208
Sedative Powers of the Root of Yellow Jessamine,.........................221
Ulceration of the Legs,................................................  241
Catherization of the Air-Passages,.......................................365
On the treatment of the more common forms of Skin Diseases met with in
the city of Edinburgh,.................................................370
United States Marine Hospital,...........................................375
Foreign Correspondence,..................................................409
Hemoptysis as a sign of Tubercle—Curability of Consumption,..............413
The Late Dr. Elisha Bartlett,............................................417
Glass Brushes for applying Fluid Caustics,...............................421
Observations on Scarlatina,..............................................456
On Digitalis,............................................................460
Local Lesions of Fever,..................................................468
Quinia as a Prophylactic,................................................469
Eczema,..................................................................469
Nitrate of Silver and Potassa,...........................................470
Yellow Ye ver,...........................................................470
Preserving Ergot,........................................................471
New Source of Alcohol,...................................................472
Causes of Headache,......................................................472
On Sterility, treatment and cure,.......................................504-
Foreign Correspondence,..................................................510
Effects of Poison in the treatment of certain Affections,................515
On the Treatment of Inflamed Breasts,....................................516
BOOK NOTICES:—
Experimental Researches,..................................................33
Diseases and Injuries of Seamen,.........................................127
Transactions of the American Medical Association,........... ............131
Fourth Annual Report of the Trustees of the Illinois State Hospital for
the Insane...............................................................171
Reports of the Trustees and Superintendent of the Butler Hospital for the
Insane,................................................................171
Nineteenth Annual Report of the Managers of the New York Institution
for the Blind,...........................................................171
Physician’s Report on Closing the Roper Hospital after,the Yellow Fever
Epidemic of 1854,......................................................161
Sanitary Reports of tho City of Buffalo for the Year 1854,.............. 171
A Manual of Pathological Anatomy,........................................177
’Transactions of the New Hampshire Medical Society,......................179
Cases of Polypus of the Womb,............................................221
Lectures in Reply to the Croonian Lectures for 1854,.....................221
Oration before the Physico-Medical Society of New Orleans,...............221
Dr. John H. Griscom’s Anniversary Discourse for 1854,....................221
Report of the Pennsylvania Hospital for the Insane, for the year 1854,... .221
Twelfth Annual Report of the Managers of the State Lunatic Asylum,
New York,..............................................................221
An Inquiry into the Pathological Importance of the Ulceration of the Os
Uteri,................................................................ 223
A Systematic Treatise, Historical, Eliological and Practical, on the Princi-
pal Diseases of the Interior Valley of North America, &c., &c..........227
Report of the Sanitary Commission on the Epidemic Yellow Fever of 1833, 324
Needs-, Duties and Privileges of the Medical Profession,.................329
Introductory Lecture to the Class of 1855, of the Medical Department of
the University of Missouri,............................................329
Valedictory Address to the Graduates of the same Class,..................329
Analysis of Blandon Springs,.............................................329
Thirty-eighth Annual Report on the state of the Asylum for the Relief of
Persons Deprived of the use of their Reason,...........................329
A Practical Treatise on Foreign Bodies in the Air Passages,..............332
A Practical Treatise on the Diseases peculiar to Woman,..................332
History of the American Medical Association,.............................377
PAGE.
The Pathology of Leucorrhoea,..........................................378
An Essay to pr'ove the Contagious Character of Malignant Cholera, with
brief Instruction for its Prevention and Cure,.........................382
Manual of Human Microscopic Anatomy,....................................383
Proceedings of the Connecticut Medical Society for 1855,................422
“Search out the Secrets of Nature.”.....................................423
A Practical Treatise on the Diseases, Injuries and Malformations of the
d1'Urinary Bladder, the prostate Gland and the Urethra,,...............423
Report of the Executive Committee of the American Temperance Union,
1855,..................................................................425
The’Indiana Journal of Medicine and	Surgery,............................425
American Journal of Pharmacy,...........................................47g
The Half-Yearly Abstract of the Medical	Sciences,.......................473
Transactions of the Medical Association of Southern Central New York,.. .473
Braithwaite’s Retrospect of Practical Medicine and Surgery,............474
Discovery of the Cause, Nature, Cure and Prevention of Epidemic Cholera, 474
Diseases of the Rectum,................................................517
A Practical Treatise on Diseases of the Eye,............................765
Yellow Fever considered in its Historical, Pathological and Therapeutical
relations,.............................................................565
The Cause and Prevention of Yellow Fever contained in the report of the
Sanitary Commission of	New Orleans,.................................566
Professor Andrews,...................................................  567
History of Medicine from its origin to the nineteenth century,.........567
Books Received,.....................................>..............569-570
MEDICAL NEWS
Hendricks County Medical Society, Ind.,................................ 38
Proceedings of the JEsculapian Society,................................321
EDITORIAL
Credit to whom Credit is Due,.......................................... 41
Sugar-house Remedy for Consumption,.................................... 45
Pumpkin Seeds for Tape Worm,.....................:......................46
Honors Properly Bestowed,...............................................47
Acne,.................................................................  47
The New Volume,.........................................................47
Prof. Brainard’s Prize Essay,..........................................136
Homoeopathy and the University of Michigan,............................138
Statistics of Mortality,.............................................    139
Rush Modical College Commencement,.....................................141
“Our EasyChair,”.......................................................143
Foreign Honors,. ’.....................................................143
History of the American Medical Association,...........................144
Index to Volume III.,..................................................144
The American Medical Association,......................................180
Convention of Delegates from Medical Colleges,.........................181
Cook County Medical Society—Variola,...................................182
Decrease of Medical Students,..........................................186
Changes Editorial,.....................................................188
Changes Professional,..................................................188
To Our Subscribers,....................................................189
To Contributors,.......................................................189
College Vacancies,.....................................................190
Health Officers,....................................................... 190
Total Abstinence and Phthisis,.........................................190
Fusel Oil in the Treatment of Phthisis,................................190
Cubebs in Incontinence of Urine in Infants,............................191
Fissures and Excoriations of the Nipple,...............................191
Orchitis, or Swelled Testicle,.........................................192
Intense Cold as an Anoesthetic,........................................192
Hospital for Females,..................................................192
PASS.
Editorial Correspondence by H. A. J.,....................................228
On the Manner in which Small Corpuscles pass from the Intestines to the
Interior of the Chyliferous and Sanguineous	Vessels,...................230
Advertising and the New York University,.................................232
The Medical Department of the University of	Michigan,....................234
Miscellaneous Items,.....................................................236
Curability of Cancer,....................................................236
Application of Caustics to Cancer,.......................................237
Congelation in Cancerous Returns,........................................337
Fissures of the Anus,....................................................237
Nocturnal Seminal Emissions,.............................................237
Scrofulous Intolerance of Light,.........................................238
University of Nashville,.................................................238
Delegates to the American Med. Association,..............................238
“Punch on Quackery,”.....................................................238
Physic and Music,........................................................239
Coesarian Section thrice upon the same Woman,............................239
Delirium Tremens. Tarter Emetic. Ovarian Dropsy. Iodine,.................240
Gonorrhoea. Subnitrate of Bismuth,.......................................240
American Medical Association,............................................254
American Medical Association,............................................296
To Our Subscribers,......................................................333
Illinois State Medical Society,..........................................334
Amputation Case,.........................................................337
Professor of Surgery in the University of Pennsylvania,..................339
Rush Medical College,....................................................340
Diseases of the Eye,.....................................................341
Miscellaneous Practical Items,...................................342-343-344
Clinical Remarks on Dysentery,...........................................385
Homoeopathy,.............................................................425
Medical Department Uuiversity of Michigan and the Peninsular Journal,. .428
Fee Bill,................................................................437
Obituary,................................................................438
Camman's Stethescope,....................................................477
Tilden & Co.’s Extracts. Rush Mecical College............................478
“Why is Norfolk Sickly?”.................................................479
Medical Education,.......................................................480
To Subscribers,..........................................................483
Toast,...................................................................484
Injections into the Bronchial Tubes and into Tubercular Cavities, of Solu-
tion of Nitrate of Silver,.............................................484
Notice,..................................................................525
Dr. Geo. Coatsworth,.....................................................525
Yellow Fever at Norfolk, Va.,............................................525
The Medical Department of the University of Michigan and the Peninsular
Journal Again,............................1............................528
Card of the Committee on Prize Essays of the American Med. Association, 570
Editorial Communication, by	A.	J.	Senimes,	M. D.,......................571
To Subscribers,..........................................................572
Personal Matters,........................................................572
Rusli Medical College,...................................................575
Professional Changes,....................................................575
What is thought of Our National Organization on the other side of the
Atlantic,..............................................................576
Number of Students in some	of	the Eastern	Med.	Colleges,..............579
Antidote for Strychnine,.................................................578
RECEIPTS UP TO JANUARY 1.
Dr J WooslWooth, Ill .	.	2 00
T Leeds	do	. 5 00
E E Elbet	do	. 2 (.0
W A Hillis, do	2 00
Jo<eph Fitch, do ,	3 10
Dr J B Guler. Wis	.	.	1 00
E Lewis. do	0 00
S B Ilnrrltnan lorn	„	.	2 00
W D Nt 1st n do	,	2 00
J K Mil*, In I.	2 00
				

